# A Rac1/Cdc42 GTPase-Specific Small Molecule Inhibitor Suppresses Growth of Primary Human Prostate Cancer Xenografts and Prolongs Survival in Mice

**DOI:** 10.1371/journal.pone.0074924

**Published:** 2013-09-11

**Authors:** Karin Zins, Trevor Lucas, Patrick Reichl, Dietmar Abraham, Seyedhossein Aharinejad

**Affiliations:** Laboratory for Cardiovascular Research, Medical University of Vienna, Vienna, Austria; Queensland University of Technology, Australia

## Abstract

Deregulated Rho GTPases Rac1 and Cdc42 have been discovered in various tumors, including prostate and Rac protein expression significantly increases in prostate cancer. The Rac and Cdc42 pathways promote the uncontrolled proliferation, invasion and metastatic properties of human cancer cells. We synthesized the novel compound AZA1 based on structural information of the known Rac1 inhibitor NSC23766. In the current study we investigated the effects of inhibition of these pathways by AZA1 on prostate tumorigenicity by performing preclinical studies using a xenograft mouse model of prostate cancer. In androgen-independent prostate cancer cells, AZA1 inhibited both Rac1 and Cdc42 but not RhoA GTPase activity in a dose-dependent manner and blocked cellular migration and proliferation. Cyclin D1 expression significantly decreased following Rac1/Cdc42 inhibition in prostate cancer cells. AZA1 treatment also down-regulated PAK and AKT activity in prostate cancer cells, associated with induction of the pro-apoptotic function of BAD by suppression of serine-112 phosphorylation. Daily systemic administration of AZA1 for 2 weeks reduced growth of human 22Rv1 prostate tumor xenografts in mice and improved the survival of tumor-bearing animals significantly. These data suggest a role of AZA1 in blocking Rac1/Cdc42-dependent cell cycle progression, cancer cell migration and increase of cancer cell apoptosis involving down-regulation of the AKT and PAK signaling pathway in prostate cancer cells. We therefore propose that a small-molecule inhibitor therapy targeting Rac1/Cdc42 Rho GTPase signaling pathways may be used as a novel treatment for patients with advanced prostate cancer.

## Introduction

Prostate cancer is the leading cause of cancer and second leading cause of cancer-related deaths in men [1]. Although screening for prostate cancer has improved, 10–20% of patients will be diagnosed with locally advanced or metastatic disease while others will progress despite surgery, radiation and androgen-deprivation therapy [2], [3]. Consequently, advanced prostate cancer remains a significant health care problem and the identification of novel targeted therapies focusing on molecular signaling pathways are essential to improve therapeutic intervention.

Rho GTPases such as Rac, Cdc42 and RhoA are signaling proteins that regulate cytoskeleton organization, cell cycle progression, cell survival and migration directly contributing to tumor growth and progression [4]. Cdc42 additionally plays a major role in the control of cell migration [5]. Rho GTPases exist either as inactive, GDP-bound forms or active, GTP-bound forms that determine the cellular functions of Rho GTPases [6]. Rho GTPase activity might be affected by differential activation of Rho GTPase regulating signaling pathways or varying amounts of Rho GTPase regulatory molecules such as Rho GTPase-activating guanine nucleotide exchange factors (GEFs) [4]. Deregulated Rho GTPases have been discovered in various proliferative malignancies, including prostate tumors [4] and Rac protein expression is significantly increased in prostate cancer [7].

Rho GTPases regulate the cell cycle by activation of c-Jun N-terminal kinase (JNK) and p38 mitogen-activated protein kinase (MAPK), leading to upregulation of cyclin D1 [8], [9], [10]. The Rac1 signaling pathway also plays a significant role in cell survival involving v-akt murine thymoma viral oncogene homolog 1 (AKT) kinase [11]. AKT in turn phosphorylates BCL2-associated agonist of cell death (BAD) [12], a proapoptotic member of the Bcl-2 family, neutralizing the proapoptotic effects of BAD [13]. p21 protein (Cdc42/Rac)-activated kinase 1 (PAK1) is a further major downstream effector of Cdc42 and Rac1 [14], [15] in the control of programmed cell death [16].

Since recent studies have implicated aberrant Rac1 and Cdc42 activity in human cancer, these Rho GTPases have been proposed as anticancer targets [17], [18]. Rac1 was one of the first targets in rational drug design approaches and a small molecule inhibitor (NSC23766) that interferes with the interaction of Rac1 with several GEFs was identified that only minimally affected Cdc42 activity [19].

We were interested in developing more potent, novel, chemically modified small molecules to inhibit Rho GTPase activity for possible clinical application in cancer treatment using the Rac1 inhibitor NSC23766 as a lead structure for compound design [19]. We now report the identification and biological activity of AZA1, a novel dual Rac1 and Cdc42 inhibitory compound that retards prostate cancer growth effectively in a human prostate cancer xenograft model.

## Materials and Methods

### Cell lines

Human androgen-independent prostate cancer cells 22Rv1 (CRL-2505), PC-3 (CRL-1435) and DU 145 (HTB-81) were obtained from American Type Culture Collection (ATCC; Manassas, VA) and cultured in Dulbecco's modified Eagle's medium (DMEM, PAA, Pasching, Austria) supplemented with 10% fetal calf serum (FCS; PAA), 0.1 M nonessential amino acids, 100 U/ml penicillin and 100 µg/ml streptomycin. Cell lines were tested for authenticity by using STR-PCR (PowerPlex 16 HS System, Promega, Madison, WI).

### Compound generation

Based on the available structural and functional information on Rac1-GEF interaction of the Rac1 inhibitor compound NSC23766 [19] and utilizing a virtual screening strategy using the ZINC database [20], we generated 21 chemically diverse potential Rac-inhibiting compound formulas, which were then synthesized by SPECS (Delft, Netherlands). Subsequently, all synthesized compounds were tested *in vitro* by solubility examination, activation assays and mitochondrial toxicity assays (WST-1) as outlined below.

### Rac1, Cdc42 and RhoA activation assays

Prostate cancer cells were seeded in 6-well plates and starved for 24 h. Cells were incubated with small molecule inhibitor AZA1 20 µM for 60 min and then stimulated with 50 ng/ml epidermal growth factor (EGF; R&D systems, Minneapolis, MN) for 90 sec and Rac1, Cdc42 and RhoA activity was then measured with G-LISA (colorimetric format, Cytoskeleton, Denver, CO) according to the manufacturer’s protocol.

### Visualization of the actin cytoskeleton and fluorescence microscopy

Human 22Rv1, DU 145 and PC-3 cells were grown on chambered coverglass in culture medium and were incubated with 50 ng/ml EGF 5 and 10 µM AZA1 for 24 h in the absence of serum. Cells were then fixed, permeabilized, labelled with Atto 488 phalloidin (Sigma-Aldrich, St. Louis, MO) and counterstained with 4’,6-Diamidino-2-Phenylindole, Dihydrochloride (DAPI, Invitrogen). Fluorescence was observed with a Nikon Eclipse 80i (Tokyo, Japan) microscope equipped with DAPI and Fluorescein-isothiocyanate (FITC) filters at 1,000x magnification and images were digitally acquired.

### Cell proliferation assay

Human 22Rv1, DU 145 and PC-3 cells were seeded in 96-well plates at a density of 1×10^4^ cells/well in culture medium. Cells were starved for 24 h and then incubated with or without 50 ng/ml EGF and 2, 5, or 10 µM AZA1. Cell proliferation was determined at 24, 48 and 72 h after treatment using the WST-1 reagent (Roche Diagnostics, Indianapolis, IN) according to the manufacturer’s protocol [21]. Each experiment was repeated three times.

### Migration assay

Prostate cancer cells (5×10^4^ in 1 ml DMEM with 10% FCS) were added to the top of each Boyden migration chamber (8-µm, 12-well plate format; BD Biosciences, Palo Alto, CA). Cells were starved for 24 h and then incubated with 50 ng/ml EGF and 2, 5 and 10 µM of AZA1. After 24 h, the medium was removed and membranes were washed twice with phosphate buffered saline (PBS). Cells from the upper side of the membrane were removed with cotton swabs. The membranes were excised using a scalpel, inverted and transferred to a PBS filled tissue culture well. Membranes were then fixed in methanol for 10 min at –20°C. After washing in PBS, membranes were stained with 1 µg/ml DAPI in PBS for 10 min at room temperature and washed again in PBS. Membranes were then embedded in Cityfluor (Cityfluor, Leicester, UK) on glass slides. Representative sectors of migrated prostate cancer cells were counted under a fluorescence microscope. Each experiment was performed in triplicate.

### FACS analysis

Tumor cells were seeded in 10 cm plates and allowed to adhere before treatment with AZA1. One portion of the cells was treated with 10 µM AZA1 for 24 h, trypsinized, washed with PBS, fixed in 70% ethanol for 1 h at 4°C, washed with PBS and stained with propidium iodide (PI) buffer supplemented with 50 µg/ml DNase-free RNaseA. Different cell cycle stages were then determined. The rest of the cells was treated with 10 µM AZA1 for 60 min before trypsinization and washing with PBS and then fixed with Cytofix fixation buffer (BD Biosciences) for 30 min at 37°C, washed and then permeabilized with Perm buffer III (BD Biosciences) and stained with Cyclin D1 (anti-human Cyclin D1 antibody set). 10^4^ events were analyzed on a FACScan flow cytometer (BD Biosciences) with an argon laser tuned to 488 nm.

### Measurement of F/G actin ratio

Prostate cancer cells were seeded in 10 cm plates and starved for 24 h. Cells were incubated with 50 ng/ml EGF (R&D systems) and 2, 5 or 10 µM AZA1 for 24 hours and levels of F-actin were then measured with the G-actin/F-actin In Vivo Assay kit (Cytoskeleton Inc., Denver, CO) according to the manufacturer’s protocol. Briefly, adherent cells were scraped and homogenized in lysis and F-actin stabilization buffer. Unbroken cells were removed by centrifugation at 350xg for 5 min. F-actin was then pelleted by centrifugation at 100,000xg for 60 min at 37°C. F-actin in the pellet and G-actin in the supernatant were analyzed by Western blotting with anti-actin antibody. Western blots were scanned using FUSION-FX7 (Vilber Lourmat, Marne-la-Vallée, France) and quantified by Fusion-CAPT-Software 16.07 (Vilber Lourmat) and the F-actin to free G-actin ratio was calculated. Each experiment was performed in triplicate.

### Western blotting

Prostate cancer cells were seeded in 10 cm plates, starved and treated with 2, 5, and 10 µM AZA1 for 24 h. Then cells were stimulated with 50 ng/ml EGF for 90 sec, lysates were prepared [22], [23] and 50 µg/lane separated by 12% SDS-PAGE prior to electrophoretic transfer onto Hybond C super (Amersham Pharmacia Biotech, Buckinghamshire, UK). The blots were probed with antibodies against phospho-PAK1 (pS144)/PAK2 (pS141) (Cell Signaling Technology, Danvers, MA), phospho-AKT (anti-phospho-AKT pT308 from BD Biosciences) and phospho-BAD (anti-phospho-BAD Ser112 from Cell Signaling Technology) before incubation with horseradish peroxidase–conjugated secondary antibodies (Amersham Pharmacia Biotech). Proteins were immunodetected by chemiluminescence (Supersignal-West- Femto, Pierce, Rockford, IL), scanned using FUSION-FX7 (Vilber Lourmat) and quantified by Fusion-CAPT-Software 16.07 (Vilber Lourmat).

### Tumor models

The experiments performed in this study were approved by the Institutional Animal Care and Use Committee at the Vienna Medical University (66.009/0095-II/10b/2008). Pathogen-free, male, 5 week-old athymic *nu/nu* (nude) mice (Charles River, Sulzfeld, Germany) were weighed, coded and divided into experimental groups at random. Mice were anesthetized (ketamine hydrochloride/xylazine at 55/7.5 mg/kg i.p.) and 15×10^6^ 22Rv1 cells/100 µl PBS were injected s.c. into the left flank [23]. Mice bearing 22Rv1 prostate cancer cell xenografts then received daily i.p. injections of 100 µg AZA1 compound in 100 µl 30% dimethyl sulfoxide (DMSO) starting at ten days following cell cancer grafting for two weeks, control animals received 100 µl 30% DMSO (*n* = 10 for each group). Tumor volumes were calculated by a caliper every other day by using the formula length x width^2^/2. All animals were sacrificed on day 24.

### Analysis of the effects of compound AZA1 *in vivo*

On day 24, animals were sacrificed and the tumors were isolated and weighed. One portion of the tissue was processed for paraffin embedding. Paraffin-embedded serial sections were rehydrated in a graded series of alcohols and antigen retrieval performed in a microwave in 0.01 M sodium citrate (pH 6.5). Following incubation in 5% H_2_O_2_ to block endogenous peroxidase activity, proliferating cells were detected with Ki-67 (proliferation-related Ki-67 antigen; MKI67) antibody (tumor proliferation assay; Dako, Glostrup, Denmark) [22], [23]. Primary antibodies were detected by sequential incubation with appropriate biotinylated secondary antibodies (Vector Laboratories, Burlingame, CA) and peroxidase conjugated streptavidin (Dako), developed with 3, 3’-diaminobenzidine (Vector Laboratories), counterstained with haemalum, dehydrated and mounted in DPX (Merck, Darmstadt, Germany) and digitalized images were generated.

### Analysis of the effects of compound AZA1 on survival

The survival study was set for three months. Mice bearing 22Rv1 xenografts were treated with compound AZA1 for 2 weeks (*n* = 10) or 30% DMSO (*n* = 10) as described above. Animals were sacrificed when they were moribund.

### Statistical analysis

Data were tested for normality using the Shapiro-Wilk test. Groups were compared by nonparametric analysis of variance (ANOVA, Wilcoxon rank-sum test, Kruskal-Wallis test). All statistical tests were two-sided. The overall survival curves after treatment were analyzed by the Kaplan-Meier survival test. Statistical tests were performed using SAS software (version 9.1.3) and Enterprise Guide (version 4.1, SAS Institute Inc., Cary, NC). Data are expressed as means ± SD. *P* values <0.05 were considered to be statistically significant.

## Results

### AZA1 treatment inhibits Rac1 and Cdc42 activity in prostate cancer cells

**Figure 1:**
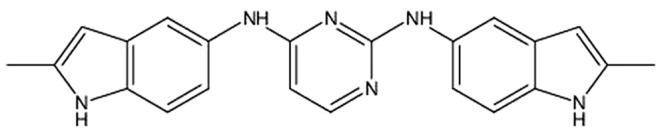
Structural formula of compound AZA1. (N*2*,N*4*-Bis-(2-methyl-1H-indol-5-yl)-pyrimidine-2,4-diamine).

**Figure 2:**
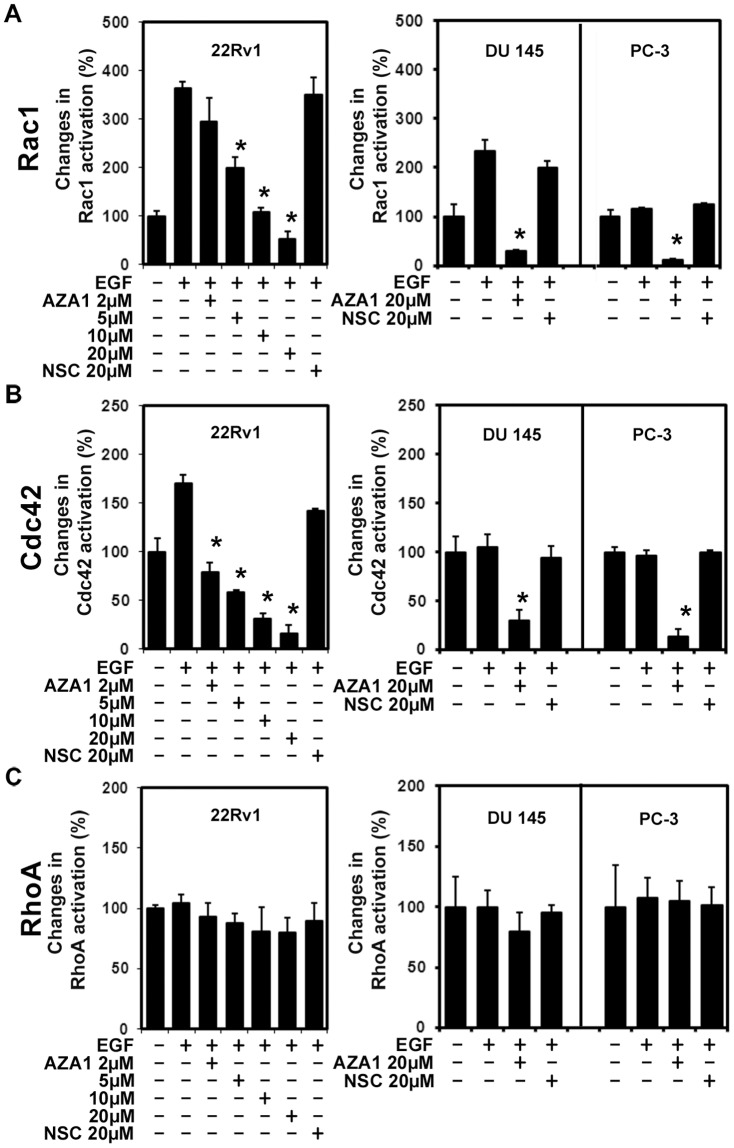
Compound AZA1 inhibits Rac1 and Cdc42 activation. **A,** Rac1 **B,** Cdc42 and **C,** RhoA activation in 22Rv1, DU145 and PC3 prostate cancer cells after incubation (60 min) with different concentrations of compound AZA1 and stimulation with 50 ng/ml EGF. Means of three independent experiments are shown. *, significantly different from EGF.

An *in vitro* screen of small molecule inhibitors based on modifications of NSC23766 to identify dual inhibitory compound activity identified the structure N*2*,N*4*-Bis-(2-methyl-1H-indol-5-yl)-pyrimidine-2,4-diamine (AZA1) (Figure 1) to have strong inhibitory activity (Text S1, Table S1, Figures S1 and S2). Initially, Rac1 activation in 22Rv1 prostate cell lysates following stimulation with EGF was examined in the screening experiments. Activation of the EGF receptor (EGF-R) plays a key role in the proliferation and invasion of prostate cancer and therefore constitutes a pathologically relevant factor for analysis of prostate cancer growth in the 22Rv1 model [24]. The activity of Rac1 was up-regulated over threefold after stimulation of 22Rv1 prostate cancer cells with EGF when compared to untreated cells. The most effective Rac1 inhibitor identified was AZA1 (Table S1). Treatment of 22Rv1 human prostate cancer cells with 5, 10 or 20 µM AZA1 for 60 min dose-dependently reduced Rac1 activity significantly by 45% (p<0.022), 70.4% (p<0.004) and 85.7% (p<0.002), respectively, compared to 20 µM NSC23766 (Figure 2A left panel). AZA1 (20 µM) also significantly down-regulated Rac1 activity in DU 145 and PC-3 prostate cell lines by 86.8% (p<0.006) and 89.9% (p<0.001), respectively (Figure 2A right panel). In addition, AZA1 treatment of 22Rv1 at 2, 5, 10 or 20 µM suppressed Cdc42 activity by 54%, (p<0.02), 65.4% (p<0.01), 81.6% (p<0.002) and 90.3% (p<0.001), respectively (Figure 2B left panel). AZA1 (20 µM) also significantly down-regulated Cdc42 activity in DU 145 and PC-3 prostate cell lines by 71.1% (p<0.0015) and 86% (p<0.007), respectively (Figure 2B right panel). In contrast, AZA1 treatment (20 µM) caused no suppression of RhoA activity (Figure 2C). These results indicate that AZA1 specifically and significantly down-regulates Rac1 and Cdc42 activity in the 22Rv1, DU 145 and PC-3 human prostate cell lines.

### AZA1 blocks the proliferation of human prostate cancer cells

**Figure 3:**
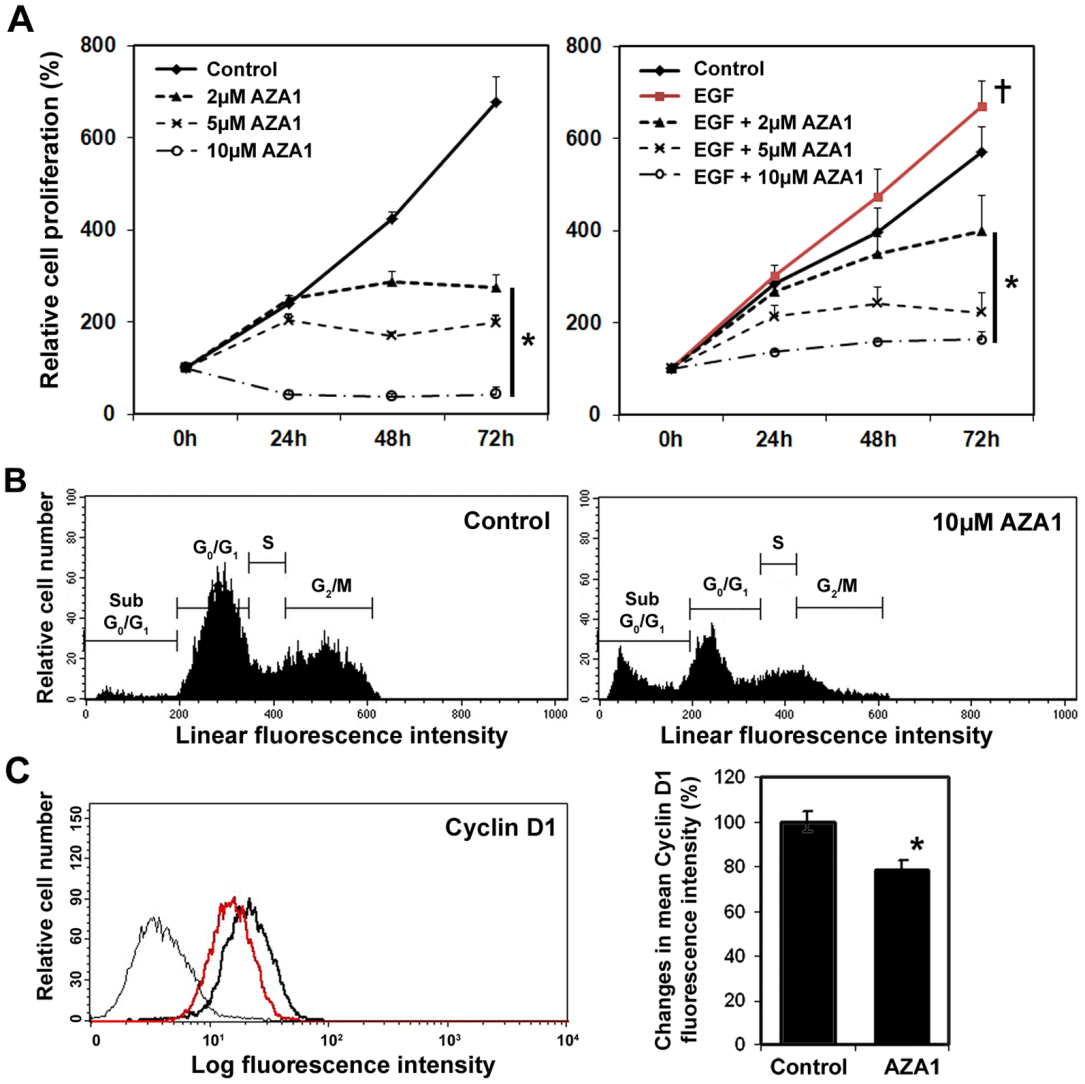
Effects of Rac1 and Cdc42 inhibition by AZA1 on cell proliferation in 22Rv1 prostate cancer cells. **A,** Relative density of cancer cells up to 72 h following treatment with 2, 5, and 10 µM compound AZA1 in unstimulated (left panel) or EGF-stimulated (right panel) cancer cells was measured using the WST-1 cell proliferation assay. AZA1 suppresses 22Rv1 prostate cancer cell proliferation in both unstimulated and EGF-stimulated cancer cells in a dose-dependent manner. Means of three independent experiments are shown. *, significantly different from control (left panel) and from control and EGF-stimulated cells (right panel); **+**, significantly different from control (right panel);. **B,** Representative flow cytometry histograms showing cell populations in sub- G_0_/G_1_, G_0_/G_1_, S and G_2_/_M_ phases. 22Rv1 cells were incubated with 10 µM AZA1 for 24 h. Control cells received no treatment. Cellular DNA content was analyzed by flow cytometry after staining with propidium iodide. **C,** Cyclin D1 expression. Representative flow cytometry analysis and quantification of fluorescence intensity in 22Rv1 cells treated with 10 µM AZA1 for 60 min (red histogram) compared to untreated cells (bold line) and isotype controls (thin line). Compound treatment reduced Cyclin D1 levels. *, significantly different vs. control.

We then analyzed the effects of AZA1 on cellular proliferation and apoptosis in target cells. Treatment of unstimulated 22Rv1 cells with AZA1 dose-dependently significantly reduced cellular proliferation after 72 h incubation with 2, 5, or 10 µM (p<0.001) AZA1 compared to control cells (Figure 3A, left panel). To analyze whether AZA1 can also reduce cellular proliferation in EGF stimulated cells, we treated EGF-stimulated prostate cancer cells with AZA1. Treatment with 50 ng/ml EGF increased 22Rv1 (p<0.05; Figure 3A, right panel), and slightly increased DU 145 and PC-3 (Figure S3) cellular proliferation. AZA1 treatment dose-dependently, significantly reduced cellular proliferation after 72 h incubation with 2, 5, or 10 µM (p<0.001) AZA1 compared to EGF-stimulated and untreated 22Rv1 (Figure 3A, right panel), DU 145 and PC-3 cells (Figure S3).

Next we analyzed cell cycle distribution following AZA1 treatment. 22Rv1 cells were separated into sub G_0_/G_1_, G_0_/G_1_, S and G_2/M_ phases. The sub G_0_/G_1_ population was used to estimate apoptosis. Figure 3B shows a representative set of data from untreated and AZA1-treated 22Rv1 cells. 22Rv1 cells treated with 10 µM AZA1 for 24 h showed an increase in the sub G_0_/G_1_ phase from 1.47% to 26.9% (±2.78; P<0.05) and a decrease in the G_2/M_ phases from 32.25% to 20.30% (±3.56; P<0.05) in cells treated with 10 µM AZA1, suggesting inhibition of cellular proliferation and an increase in the number of apoptotic events. We then tested the effect of AZA1 on the cell cycle regulatory protein Cyclin D1. In cell lysates treated with 10 µM AZA1 for 60 min, Cyclin D1 fluorescence intensity decreased significantly by 22%±4.2% (p<0.001) compared to untreated controls representatively shown in Figure 3C. These results indicate a role for AZA1 in blocking Rac1 and Cdc42-dependent cell cycle events in 22Rv1 prostate cancer cells and induction of apoptosis.

### AZA1 inhibits cancer cell migration

**Figure 4:**
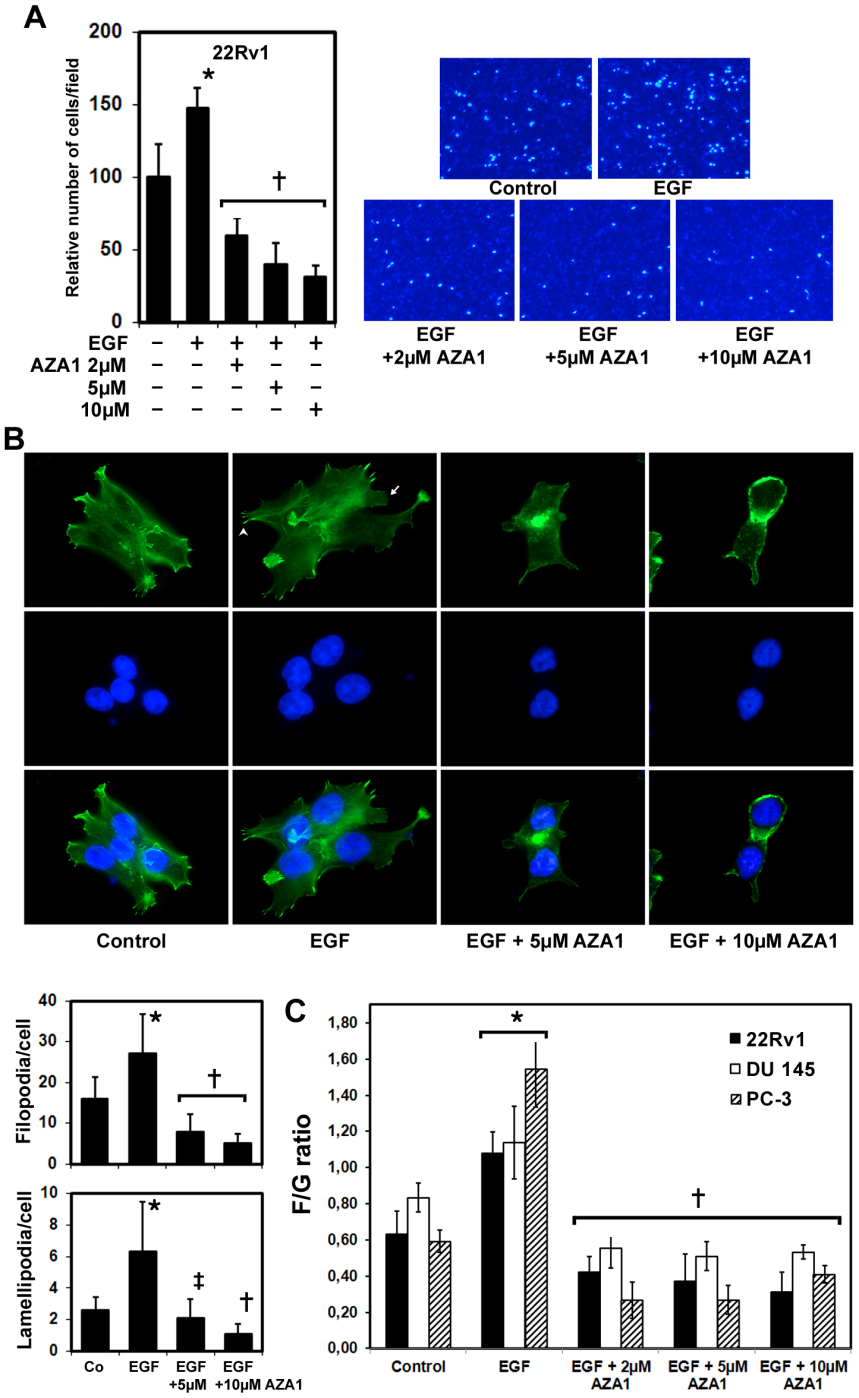
Rac1 and Cdc42 blockade reduces prostate cancer cell migration and affects cytoskeletal dynamics. **A,** Representative images of migrated prostate cancer cells from an *in vitro* migration assay are shown. 22Rv1 prostate cancer cells were stimulated with 50 ng/ml EGF and treated with 2, 5 or 10 µM AZA1 for 24h and migrated cancer cells quantified subsequently in *in vitro* migration assays. Data were collected from five individual consecutive fields of view (40x) from three replicate Boyden chambers. *, significantly different from control; **+**, significantly different from control and EGF-stimulated cells. **B,** Effect of AZA1 treatment on lamellipodia and filopodia formation. 22Rv1 prostate cancer cells were plated on cell culture chambers, stimulated with 50 ng/ml EGF and incubated with 5 and 10 µm AZA1 for 24 h. Paraformaldehyde fixed cells were stained with Atto-488 phalloidin (F-actin, green) to detect polymerized actin cytoskeleton, filopodia and lamellipodia and counterstained with DAPI (blue) and photographed (magnification, x1000). Arrow head indicates filopodia, arrow indicates lamellipodia. The numbers of filopodia and lamellipodia per cell were calculated from 25 cells in each group. AZA1 leads to changes in cellular morphology and suppresses filopodia and lamellipodia formation. *, significantly different from controls; **+**, significantly different from controls and EGF-stimulated cells; **‡**, significantly different from EGF-stimulated cells. Co, control. **C,** Effect of AZA1 on actin dynamics. Expressions of F-actin and G-actin in 22Rv1, DU 145 and PC-3 cells analyzed by immunoblotting (F-actin/G-actin ratio). Bars represent the F/G actin mean value ±SD. *, significantly different from controls of the respective cell line; **+**, significantly different from controls and EGF-stimulated cells of the respective cell line.

Active migration of tumor cells is a prerequisite for tumor progression and metastasis and reports show that EGF-induced cancer cell migration can be inhibited by suppressing Cdc42/Rac1 signaling pathways [25]. Thus, we examined the effect of AZA1 on the migration of EGF-stimulated 22Rv1, DU 145 and PC-3 cells in Transwell assays. EGF-stimulation significantly increased cancer cell migration in 22Rv1 (Figure 4A), DU 145 and PC-3 (Figure S4A) cells (p<0.001). Treatment of cells with 2 µM AZA1 for 24 h significantly reduced cancer cell migration by 59.6 ±12% (22Rv1 cells; p<0.001; Figure 4A), 56.8 ±18.8% (DU 145 cells; p<0.001; Figure S4A) and 57.3 ±16.1% (PC-3 cells; p<0.001; Figure S4A) compared to EGF-stimulated cancer cells.

Treatment with 2 µM AZA1 also reduced cancer cell migration in all three cell lines when compared to control cells without EGF-stimulation by 40.2 ±12.1% (22Rv1 cells; p<0.01; Figure 4A), 20.2 ±8.9% (DU 145 cells; p<0.04; Figure S4A), and 24.9±16.1% (PC-3 cells; p<0.05; Figure S4A), respectively compared to EGF-stimulated cancer cells.

Treatment of cells with 5 µM and 10 µM AZA1 further reduced migration by 72.1% and 79.1% (p<0.001) in 22Rv1 cells, by 72.4% and 91.4% (p<0.001) in DU 145 cells, and by 60.9% and 74.7% (p<0.001) in PC-3 cells, respectively, compared to EGF-stimulated cells (Figures 4A and Figure S4A). These results indicate a role for AZA1 in blocking Rac1 and Cdc42-dependent migration of 22Rv1, DU 145 and PC-3 prostate cancer cells.

### AZA1 reduces lamellipodia and filopodia formation and decreases the F/G-actin ratio

Cdc42 and Rac1 are also crucial in the formation of filopodia and lamellipodia, which are important in the invasion of cancer cells [26]. We therefore investigated the effect of AZA1 on cell morphology using phalloidin staining of 22Rv1, DU 145 and PC-3 cells that specifically stains polymerized actin cytoskeleton. Morphologically, the numbers of 22Rv1 lamellipodia and filopodia significantly increased on EGF-stimulation (p<0.04; Figure 4B). Treatment with AZA1 at 5 and 10 µM resulted in significantly reduced lamellipodia (p<0.01) and filopodia (p<0.01) formation in 22Rv1 prostate cancer cells after 24 h compared to EGF-stimulated cells (Figure 4B).

In DU 145 cells, whereas numerous filopodia are observed that increase following EGF-stimulation, almost no lamellipodia are visible. Similar to the effect on 22Rv1 cells, treatment with AZA1 at 5 and 10 µM resulted in dramatically reduced filopodia formation in DU 145 prostate cancer cells after 24 h compared to EGF-stimulated cells (Figure S4B, upper three panels). In PC-3 cells, both lamellipodia and filopodia formation occurred in control and EGF-stimulated cells. Following treatment with AZA1 (5 and 10 µM), lamellipodia were almost undetectable and cells developed a more rounded morphology. Filopodia were still observed after 5 µM AZA1 treatment, although were reduced at 10 µM AZA1 (Figure S4B, lower three panels).

Therefore, AZA1 inhibited lamellipodia and filopodia formation in prostate cancer cells, displaying different degree of suppression depending on the cancer cell type. These data indicate a direct regulatory effect of Rac1/Cdc42 activity on lamellipodia and filopodia extension in all tested cell lines that can be affected by AZA1 treatment. Together, these data indicate a specific role for AZA1 in inhibiting Rac1/Cdc42-mediated cell morphology.

Next we examined the F- and G-actin contents in prostate cancer cells to determine whether AZA1 can affect dynamic actin rearrangement. AZA1 reduced lamellipodia and filopodia formation and reduced cancer cell migration. Since Cdc42 and Rac are known to play major roles in actin rearrangement, which is a prerequisite in these processes [28], we evaluated the effect of Rac1/Cdc42 inhibition on the relationship between Rac1 and Cdc42 activity and dynamic reorganization of the actin skeleton. Immunoblotting analyses of the F- and G-actin fractions showed that the relative expression level of F-actin/G-actin was significantly higher in EGF-stimulated 22Rv1, DU 145 and PC-3 prostate cancer cells compared to control cells (p<0.05; Figure 4C). Treatment with AZA1 at 2, 5 and 10 µM significantly reduced the relative expression ratio of F- to G-actin in all three cell lines after 24 h compared to EGF-stimulated cancer cells (p<0.01; Figure 4C) and control cells (p<0.05; Figure 4C).

These findings indicate that lamellipodia and filopodia formation in prostate cancer cells is associated with the reorganization of actin filaments and that this process is affected by AZA1 treatment.

### AZA1 down-regulates the PAK signaling pathway

**Figure 5:**
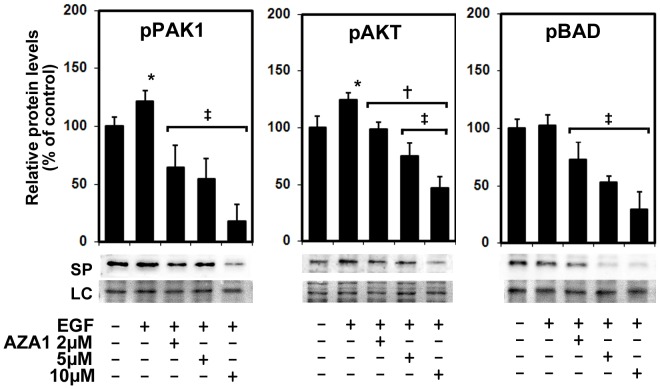
AZA1 down-regulates PAK signaling. Analysis of PAK, AKT and BAD-phosphorylation in EGF-stimulated 22Rv1 prostate cancer cells following AZA1 treatment. Representative Western blot images and quantification of immunoblots stained with phospho-PAK1/2 (pPAK), phospho-AKT (pAKT) and phospho-BAD (pBAD) antibodies before and after treatment with 2, 5 and 10 µM AZA1 for 24 h. Rac1/Cdc42 blockade reduces phosphorylation of PAK1, AKT and BAD in 22Rv1 prostate cancer cells compared to controls (means of 3 independent experiments). *, significantly different from unstimulated and untreated controls; **+**, significantly different from EGF-stimulated control; **‡**, significantly different from unstimulated, untreated control and EGF-stimulated control. SP, specific protein; LC, loading control.

Group I p21-activated kinases (PAK) are important effectors of the small GTPases Rac and Cdc42, which regulate cell migration, survival and proliferation [27], [29]. PAK also regulates Rac-mediated lamellipodia extension, migration and invasion of cancer cells [27]. To analyze signaling pathways that could mediate the effects of AZA1 mediated Rac1/Cdc42 inhibition, we examined the activity of the downstream effector PAK by evaluating PAK phosphorylation in EGF-stimulated cancer cells following AZA1 treatment. The data show that PAK1/2 phosphorylation at serine 144/141, which maintains the catalytic activity of PAKs [30], was dose-dependently significantly reduced by 46.9±19.1% (2 µM), 55.5±18.4% (5 µM) and 85±14.3% (10 µM) (p<0.04) on AZA1 treatment in 22Rv1 cells compared EGF-stimulated cells (Figure 5). Similarly, PAK1/2 phosphorylation at serine 144/141 was also dose-dependently and significantly reduced up to 52.4±15.1% (p<0.05) and 48.1±11.5% (p<0.04), respectively, on AZA1 treatment of EGF-stimulated DU 145 and PC-3 cells (Figure S5), indicating that Rac1/Cdc42 inhibition blocks the PAK1 signaling pathway in these prostate cancer cells. PAK1 protein levels were not affected by AZA1 treatment (data not shown). These findings suggest that AZA1 affects cell motility and actin rearrangement in prostate cancer cells by suppressing Rac1 and Cdc42 activity via PAK1/2 phosphorylation.

### AZA1 down-regulates the AKT signaling pathway and reduces BAD phosphorylation

In order to identify further downstream Rac1/Cdc42 candidates affected by AZA1 treatment, we analyzed AKT and MAPKs activity using phospho-specific antibodies. AKT and ERK activities have been decreased by reduction of PAK1 expression leading to decreased cell proliferation, migration/invasion and survival in colon cancer [28]. Moreover, the AKT signaling pathway has been shown to be involved in prostate cancer progression and the transition to androgen-independent disease [31].

We found that Rac1/Cdc42 inhibition by AZA1 for 24 h led to a significant dose-dependent inhibition of phospho-AKT levels by 20.8% (2 µM), 39.3% (5 µM) and 62.5% (10 µM) (p<0.05) following AZA1 treatment of EGF-stimulated 22Rv1 cells (Figure 5). In contrast, AZA1 treatment caused no changes in phosphorylation of the potential Rac1 effectors ERK, JNK or p38 (data not shown), indicating that activation of these MAPK pathways is not affected by Rac1 and Cdc42 inhibition in 22Rv1 prostate cancer cells. In DU 145 cells phospho-AKT levels were also significantly reduced 8% (2 µM), 12.4% (5 µM) and 28.4% (10 µM) by AZA1 treatment (p<0.05) compared to EGF-stimulated cells, however, in contrast to 22Rv1 cells, phospho-AKT levels never decreased below the levels in untreated or unstimulated control cells. In a similar manner, while phospho-AKT levels in PC-3 significantly decreased compared to EGF-stimulated cells upon AZA1 treatment 12.4% (2 µM), 50% (5 µM) and 58.8% (10 µM) compared to EGF-stimulated cells, levels decreased below untreated and unstimulated levels in control cells at 10 µM AZA1 (Figure S5). These results suggest that a Rac1 and Cdc42 targeting strategy can effectively modulate EGF-stimulated PAK/AKT signaling pathways to affect prostate cancer cell survival and proliferative response.

The mediators of the observed effects of AZA1 on cell apoptosis remain unclear. To address this issue, we investigated BAD phosphorylation in Ser112 following AZA1 treatment, since it has been reported that regulation of prostate cancer cell survival involves BAD phosphorylation [32]. EGF-stimulated 22Rv1 cells were treated with 2, 5 and 10 µM AZA1 for 24 h and phosphorylated BAD levels were determined by Western blotting. Remarkably, AZA1 treatment led to a dose-dependent, significant reduction of BAD phosphorylation at Ser112 by 29.2% (2 µM), 48.8% (5 µM) and 71% (10 µM) (p<0.05) compared to controls (Figure 5). In DU 145 and PC-3 cells, AZA1 treatment also led to a dose-dependent significant reduction of phospho-BAD up to 35% (DU 145) and 23.8% (PC-3) at 10 µM AZA1 (p<0.05) compared to EGF-stimulated cells (Figure S5). Thus, we provide evidence for the involvement of the BAD apoptotic pathway in the response to Rac1/Cdc42 inhibition by AZA1 as dephosphorylated BAD, which induces apoptosis, is associated with signs of increased apoptosis upon AZA1 treatment.

### AZA1 suppresses primary prostate cancer growth and improves survival in mice

**Figure 6:**
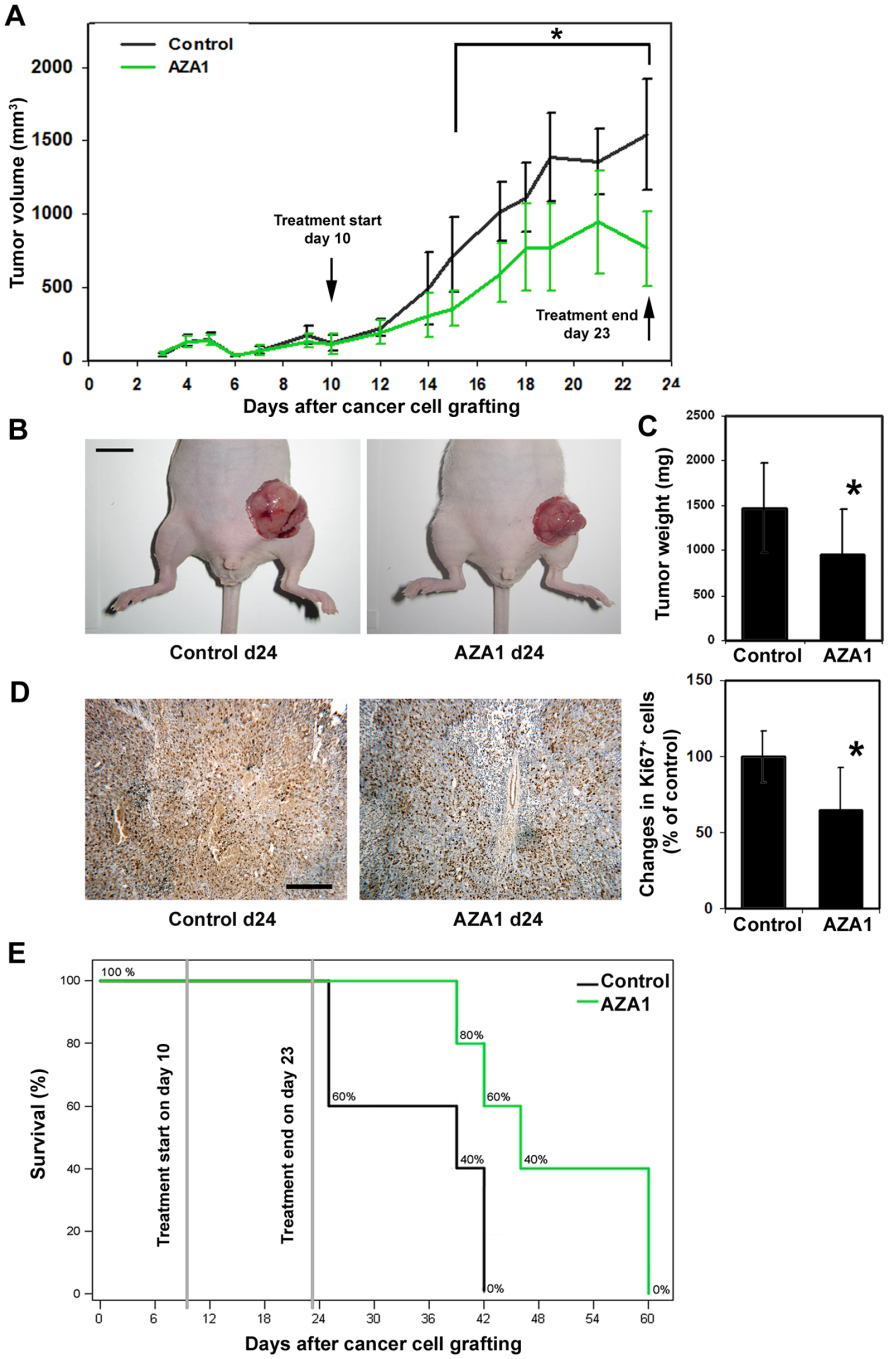
Rac1 and Cdc42 blockade suppresses tumor growth and prolongs animal survival. **A,** Tumor volume curves in 22Rv1 tumor xenograft bearing mice treated with AZA1 or solvent only (Control). *, significantly different from control. **B,** representative images of human prostate tumor xenografts on day 24 from mice treated with solvent (Control) or 100 µg/day AZA1 (AZA1 d24) (bar  = 1 cm). **C,** AZA1 significantly suppresses tumor weight of human prostate xenografts in mice. Data are shown as mean tumor weights on day 24. *, significantly different from control on day 24. **D,** left panel: Representative immunohistochemistry images of tumor tissue sections from mice treated with solvent (Control) or 100 µg/day AZA1 on day 24 stained with Ki-67 antibody (bar  = 200 µm). Cellular proliferation is reduced following Rac1 and Cdc42 blockade. Right panel: Quantitative histomorphometric analysis of Ki-67-positive, proliferating tumor cells. *, significantly different from control. **E,** effect of AZA1 treatment on animal survival in 22Rv1 prostate cancer bearing mice. AZA1 significantly prolongs animal survival vs. untreated controls.

To analyze whether treatment with AZA1 plays a role in tumor growth, we treated mice bearing human prostate cancer xenografts with AZA1 or vehicle as controls. To assess treatment modalities *in vivo*, we initially assessed AZA1 stability *in vitro* (data not shown) and cycled treatment daily for two weeks to guarantee continuous reduction of tumor cell-derived Rho GTPases. At the beginning of treatment on day 10, mice developed human tumors of comparable size. From measuring tumor volumes, the suppressive effect of AZA1 on tumor growth was significant from days 15 to 24 (p<0.05), at which time the animals were sacrificed (Figure 6A). On day 24, the mean tumor weight was markedly reduced in mice treated with AZA1 (956 mg ± 505 mg) compared to control mice (1470 mg ± 497 mg) (p<0.03) (Figures 6B, C). In accordance with these tumor weight findings, cell proliferation as assessed by Ki-67 staining was reduced following treatment with AZA1 (p<0.05) (Figure 6D), suggesting an anti-proliferative effect of Rho GTPase inhibition by the compound AZA1.

The median time to death in the control group was 39±15 days and all mice died between 25 and 42 days after tumor cell grafting. However, survival was significantly increased in mice receiving AZA1 treatment compared to controls (p<0.05) and the median time to death was 46±4 days. At day 42, at which time the last animal in the control groups died, 60% of animals treated with the AZA1 were still alive (Figure 6E).

These data indicate that AZA1 is potent in suppressing human 22Rv1 xenograft growth in mice and improving survival.

## Discussion

The molecular complexity and redundancy of signaling in prostate cancer calls for simultaneous inhibition of multiple pathways to achieve an effective treatment response [33]. In this context, Rho GTPase family activity might be altered due to deregulation of signaling pathways that are involved in proliferation, survival and response to apoptotic cell death signals [4], [34]. As no constitutively active mutant forms of Rho GTPases have been found in human tumors [4] and overexpression of Rho GTPases such as Rac1 and Cdc42 occurs in many malignancies including prostate cancer [7], [35], [36], [37], it can be assumed that over-expression rather than Rho GTPase activation mutations is associated with tumorigenesis. This fact has important therapeutic implications. Rac1, for example, is also involved in cell adhesion and migration and Rac1 activation is associated with increased tumor invasiveness [38], suggesting that Rac1 and downstream effectors are useful anticancer targets. In line with this, studies have shown that Rac inhibition by a small molecule inhibitor NSC23766 affects Rac-hyperactive PC-3 prostate cancer cell proliferation, growth and invasion [19]. Our objective was to develop a novel potent small molecule inhibitor of Rac activity based on the principal structure of known Rac-inhibiting compounds [19], [20]. Designed candidate factors were screened for down-regulation of endogenous Rac activity compared to the existing inhibitor NSC23766. We identified AZA1 as a novel Rac and Cdc42 inactivating compound with significantly improved inhibition of Rac combined with additional Cdc42 inhibitory activity when used in EGF-stimulated 22Rv1 prostate cancer cells. Our data show that 22Rv1 cancer cell growth is stimulated by EGF and tyrosine kinase receptors contribute to the androgen-independent proliferation of these cells [39]. Moreover, activation of the EGF receptor in prostate cancer contributes to metastatic progression in addition to disease relapse [40] and increased EGF receptor expression correlates with tumor recurrence, high Gleason score and advanced disease stage [41]. In this context, it is important to mention that AZA effectively reduced 22Rv1 cell proliferation in EGF stimulated as well as untreated control cells. Moreover, AZA1-treatment also resulted in Rac1 and Cdc42 inhibition associated with suppression of cell proliferation in EGF-stimulated androgen-independent cell lines DU 145 and PC-3 *in vitro*, supporting the potential of AZA1 for use in prostate cancer. Importantly, we show that 2 weeks of systemic AZA1 treatment *in vivo* selectively suppressed the growth of human 22Rv1 prostate cancer xenografts and significantly improved animal survival.

Our finding that AZA1 treatment also suppressed Cdc42 but not RhoA activity in addition to Rac activity can be explained by the fact that Rho GTPases share a substantial degree of regulatory-site structural homology. Consequently, the development of specific small-molecule inhibitors of the various Rho GTPases is a challenging task. However, since both Cdc42 and Rac1 signaling contribute to cellular transformation and tumor progression, inhibition of several GTPases could prevent bypassing of the blocked pathways and might constitute a therapeutic advantage. Cdc42 activity has also been implicated in the proliferation and invasion of colorectal cancer cells involving downstream PAK signaling, suggesting that Cdc42 may be a useful target for therapeutic intervention in addition to Rac1 inhibition [42]. Of interest, the tumor suppressor maspin has been reported to mediate tumor cell migration through inhibiting Rac1 and Cdc42, but not RhoA GTPase [29], suggesting that targeting the Rac1/Cdc42 pathways by AZA1 could counteract the activity of maspin in prostate cancer cells. On the other hand, maspin expression consistently appears to be down-regulated at the critical transition from non-invasive, low grade to high grade human prostate cancer [43]. The acquired migratory phenotype of invasive cancer cells correlates with the formation of lamellipodia and filopodia, which are formed by a highly dynamic cytoskeleton. These structures are also under the control of the Rho GTPases Rac and Cdc42 and their effector proteins [31] [44]. In this context, F-actin, which is the polymerized form of G-actin, plays a critical role in Rho GTPase-driven lamellipodia and filopodia formation in order to drive cell migration [45]. In metastatic MTLn3mammary adenocarcinoma cells stimulation with EGF results in extensive actin polymerization leading to lamellipod extension and cell motility [46], [47]. Similarly, we observed a high ratio of F-actin to G-actin in all three investigated prostate cancer cell lines, which was further increased by EGF stimulation. This stabilization of F-actin polymerization upon EGF-stimulation was associated with increased migration, lamellipodia and filopodia formation in prostate cancer cells. In previous studies in lung cancer cells, the invasion suppressor gene CRMP1 has been shown to inhibit cancer cell invasion through F-actin depolymerization and inhibition of filopodia formation, whereas the long isoform of CRMP1, promoted cell migration and filopodia formation by increasing the ratio of F-actin to G-actin [32], [39]. In our study we identified the small molecule inhibitor AZA1 as suppressing prostate cancer cell migration through decreasing F-actin to G-actin ratios and inhibiting lamellipodia and filopodia formation.

Our data show a significant reduction in phosphorylation and hence activation of AKT following Rac/Cdc42-inhibition by AZA1. The AKT signaling pathway is known for its role in mediating cell survival, as well as cell cycle progression and neoplastic transformation and appears to be critical for prostate cancer cell survival and proliferation [48], [49]. Moreover, it has been reported that the AKT signaling pathway contributes to prostate tumorigenicity and androgen independence [50]. Activated AKT translocates to the cytoplasm and nucleus and activates downstream targets involved in survival, proliferation cell cycle progression, growth and cell migration [49]. The results presented here demonstrate an inhibition of cancer cell proliferation *in vitro* and *in vivo* following Rac1/Cdc42-inhibition. In addition to downregulated AKT activity, reduced Cyclin D1 expression by AZA1 treatment fits to these findings, since evidence suggests that Rac1 affects transformation through regulation of Cyclin D1, a cell cycle protein that is frequently over-expressed in prostate cancer xenografts and metastases [51], [52]. Cyclin D1 is known to regulate the cell cycle by stimulating phosphorylation of the retinoblastoma (Rb) protein, which subsequently triggers transcription of various genes required for G1 progression [53]. Overexpression of Cyclin D1 has also been reported to increase tumorigenicity of the LNCaP prostate cancer cell line [54]. Cyclin D1 expression may be regulated in prostate cancer as a consequence of AKT-mediated activation leading to overexpression of several key proteins including Cyclin D1 [55], [56], a hypothesis, which is also supported by our data. AKT in turn can be activated by the Rac1 effector, p21 activating kinase 1 (PAK1), which activates AKT by direct interaction [15]. PAK binds Rac1 in a GTP-dependent manner, potently stimulating PAK kinase activity. PAKs are pivotal molecules for multiple signaling pathways [57] and are involved in transforming mammary epithelial cells [14]. A recent report showing that PAK1 signaling is critical to medulloblastoma cell migration [58] suggests that reduced PAK1 activity mediated through Rac1 inhibition in our prostate cancer model may also account for the suppressed cell migration observed. Cdc42 inhibition could play a major role in this effect, since Cdc42 has been shown to contribute to cell migration and invasion dependent on the cell type [59]. Since PAK1 can also regulate cell transformation and survival and because of increased frequency of phosphorylation observed in poor outcome tumors [16], the PAK signaling pathway could therefore constitute a major effector pathway of Rac1/Cdc42 in prostate cancer cells.

In support of this, our results suggest that PAK1 acts downstream of Rac and Cdc42 to promote actin polymerization, filopodia formation and cancer cell invasion, showing that F-actin reorganization plays a major role in cell movement in prostate cancer cells. Furthermore, our findings suggest that inhibition of tumor cell motility by AZA1 could contribute to the anti-cancer effects observed.

AKT phosphorylation of BAD has been shown to block BAD-induced cell death [12]. BAD function is regulated by phosporylation at serine 112 and serine 136 thereby promoting cell survival. In the absence of phosporylation of these sites, BAD is thought to induce cell death [60], [61]. The BAD protein represents a switch integrating the antiapoptotic effects of multiple pathways in prostate cancer cells, thus expression of a nonphosphorylatable mutant S112A-BAD reduces the survival effects of growth factors such as EGF in prostate cancer cells [27], [30]. This is in line with our data showing reduced phosphorylation of BAD at Ser-112 associated with increased rates of cell death. Thus, we provide evidence for the involvement of the BAD apoptotic pathway in the response to Rac1/Cdc42 inhibition by AZA1, although it remains unclear whether BAD phosphorylation is dependent of AKT in this context.

**Figure 7:**
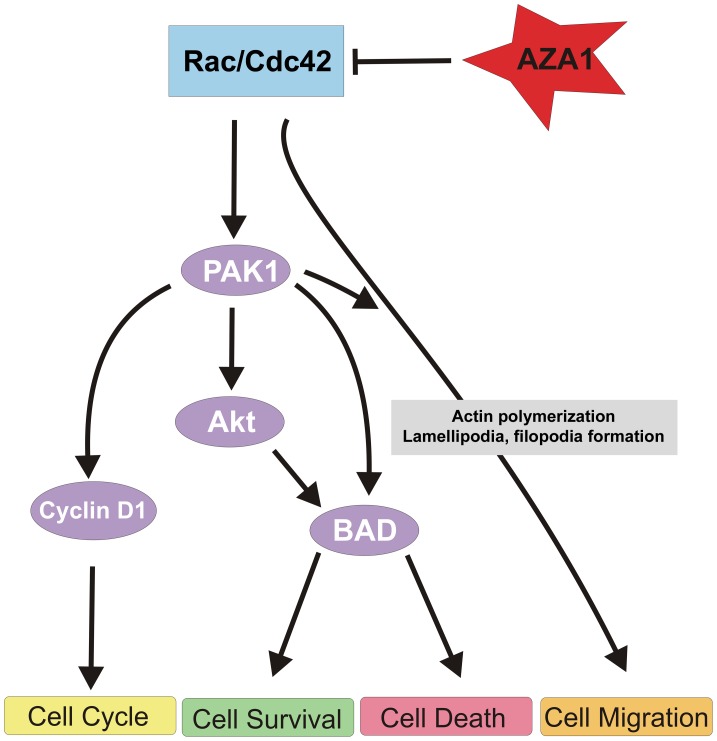
Proposed AZA1 regulated pathways downstream of Rac1 and Cdc42 in prostate cancer. AZA1 inhibits activation of Rac1 and Cdc42 GTPases, shifting the balance towards cell growth inhibition and apoptosis. These effects are exerted by different mechanisms. AZA1 inhibits the PAK pathway via the cell cycle regulator Cyclin D1 and suppresses PAK and AKT activation leading to reduced BAD phosphorylation to induce pro-apoptotic activity. In addition, suppression of Rac1 and Cdc42 suppresses cancer cell migration and invasion by affecting actin polymerization and subsequent inhibition of lamellipodia and filopodia formation.

Consequently, we hypothesize that Rac1/Cdc42 inhibition by AZA1 treatment increases cancer cell apoptosis involving the PAK-AKT-BAD signaling pathways (Figure 7). In addition, Rac1 can signal through PAK to activate JNK [62]. Although Rac1 activation was found to promote cell survival by activation of AKT, ERK and NF-κB, it can also induce apoptosis by stimulation of p38 and JNK [63]. However, in the 22Rv1 prostate cancer cells analyzed here, phosphorylation of p38 and JNK was not affected following Rac1-inhibition. Thus, the observed unchanged activity of p38 and JNK following Rac1/Cdc42-inhibition by AZA1 suggests that these pathways play no major role in mediating AZA1-induced anti-proliferative effects in 22Rv1 prostate cancer cells.

Similarly to JNK and p38, ERK activity remained unchanged in prostate cancer cells following AZA1 treatment in our studies. Comparable to these results, down-regulation of PAK1 in medulloblastoma cells did not alter platelet-derived growth factor mediated activation of ERK [58] and a study in osteoclast cells similarly showed that PAK1 does not modulate Raf-mediated MEK activation by macrophage colony stimulating factor [64]. On the other hand, although the MEK/ERK MAPK pathway is known for its role in cell proliferation, there is little evidence that MAPK activity is increased in prostate cancer and interactions between Rac1/PAK1 and MEK/ERK is likely to be cell type-dependent [65]. In addition, ERK activity was not affected by Rac1 inhibition in lymphoma cells further suggesting that this Rac1 targeting strategy exerts its effect via modulation of the PAK1-AKT signaling pathway, but not the MAP kinases, to affect prostate cancer cell proliferation [66].

Together, the present study describes a new small molecule Rac1- and Cdc42-inhibiting agent. We provide evidence that Rac1 and Cdc42 but not Rho activity is downregulated by AZA1 in prostate cancer cells and that combined Rac1 and Cdc42 inhibition suppresses cell proliferation and activation of PAK and AKT and signaling pathways affecting the downstream cell cycle regulator Cyclin D1 and the pro-apoptotic protein BAD. Additionally, inhibition of Rac1/Cdc42 by AZA1 affected cytoskeletal dynamics and suppressed cancer cell migration. AZA1 reduced tumor growth and prolonged survival in a human prostate cancer xenograft mouse model. We propose that AZA1 could serve as a dual, Rac1 and Cdc42-targeting, small molecule inhibitor in the treatment of patients with advanced prostate cancer.

## Supporting Information

Figure S1 Chemical structures of group 1 potential Rac-GTPase-inhibiting compound formulas theoretically considered for *in vitro* testing. For Materials and Methods see text S1.
(TIF)

Figure S2 Chemical structures of group 2 potential Rac-GTPase-inhibiting compound formulas theoretically considered for *in vitro* testing. For Materials and Methods see text S1.
(TIF)

Figure S3 Rac1 and Cdc42 inhibition by AZA1 reduces the proliferation of DU 145 and PC-3 prostate cancer cells. Relative density of cancer cells up to 72 h following treatment with 2, 5, and 10 µM compound AZA1 in EGF-stimulated cancer cells was measured using the WST-1 cell proliferation assay. AZA1 suppresses DU 145 and PC-3 prostate cancer cell proliferation in EGF-stimulated cancer cells in a dose-dependent manner. Means of three independent experiments are shown. *, significantly different from untreated control and EGF-stimulated cells (p<0.05).
(TIF)

Figure S4 Rac1 and Cdc42 blockade reduces prostate cancer cell migration and affects cytoskeletal dynamics in DU 145 and PC-3 prostate cancer cells. **A,** Rac1 and Cdc42 blockade reduces prostate cancer cell migration. DU 145 and PC-3 prostate cancer cells were stimulated with 50 ng/ml EGF and treated with 2, 5 and 10 µM AZA1 for 24 h and migrated cancer cells quantified subsequently *in vitro*. Data were collected from five individual consecutive fields of view (40x) from three replicate Boyden chambers. *, significantly different from control; **+**, significantly different from control and EGF-stimulated cells. **B,** Effects of AZA1 treatment on lamellipodia and filopodia formation. DU 145 (upper three panels) and PC-3 (lower three panels) prostate cancer cells were plated on cell culture chambers, stimulated with 50 ng/ml EGF and incubated with 5 and 10 µm AZA1 for 24 h. Paraformaldehyde fixed cells were stained with Atto-488 phalloidin (F-actin, green) and nuclei were counterstained with DAPI (blue). Lowest panel: merge panel. Arrow head indicates filopodia, arrow indicates lamellipodia. AZA1 leads to changes in cellular morphology and suppresses filopodia (DU 145 and PC-3) and lamellipodia (PC-3) formation (magnification, x1000).
(TIF)

Figure S5 Analysis of PAK-, AKT- and BAD-phosphorylation in EGF-stimulated DU 145 (upper panel) and PC-3 (lower panel) prostate cancer cells following AZA1 treatment. Representative Western blot images and quantification of immunoblots stained with phospho-PAK1/2 (pPAK), phospho-AKT (pAKT) and phospho-BAD (pBAD) antibodies before and after treatment with 2, 5 and 10 µM AZA1 for 24 hours. Rac1/Cdc42 blockade reduces phosphorylation of PAK1, AKT and BAD in prostate cancer cells compared to controls (means of 3 independent experiments). *, significantly different from unstimulated and untreated control; **+**, significantly different from EGF-stimulated control; ‡, significantly different from unstimulated, untreated control and EGF-stimulated control.
(TIF)

Table S1 21 synthesized compounds tested *in vitro* for solubility, GTPase activation and effects on cell proliferation. Compound AZA1 was selected for further experiments *in vivo*. Solubility: Soluble in 30% dimethyl sulfoxide (DMSO) at 1 mM compound concentration: (+), soluble; (−), insoluble or poorly soluble. Toxicity (WST-1 cell proliferation assay): (+), substance tolerable; (−), substance discarded due to toxicity at < 25 µM (concentrations tested: 1−100 µM). Rac-Inhibition: (+), (++) relative inhibition of Rac activity; (−) negligible inhibition of Rac activity. For Materials and Methods see text S1.
(DOCX)

Text S1 Supplemental Materials and Methods
(DOCX)
