# Supplementary figures and images for: A Rac1/Cdc42 GTPase-Specific Small Molecule Inhibitor Suppresses Growth of Primary Human Prostate Cancer Xenografts and Prolongs Survival in Mice
Source: PLoS One. 2013 Sep 11;8(9):e74924. doi: 10.1371/journal.pone.0074924 (PMC3770583; doi:10.1371/journal.pone.0074924)

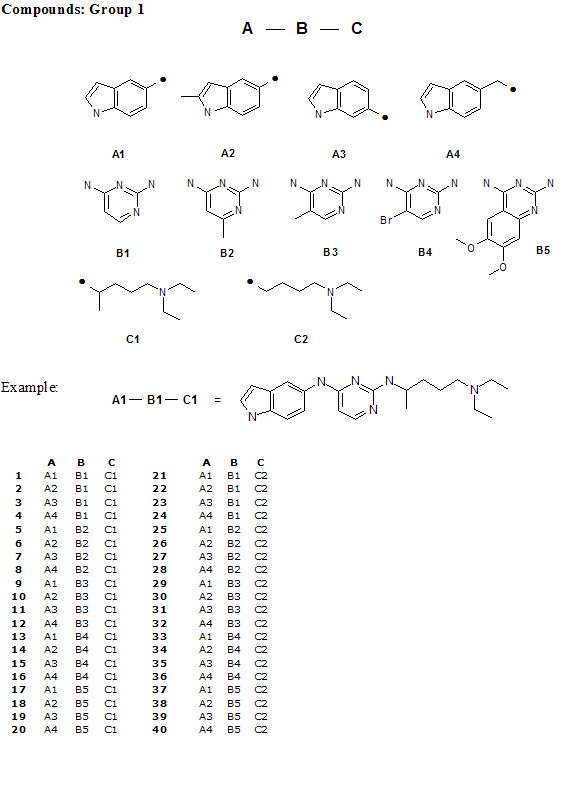

Supplement: Figure S1 — Chemical structures of group 1 potential Rac-GTPase-inhibiting compound formulas theoretically considered for in vitro testing. For Materials and Methods see text S1. (TIF) [file pone.0074924.s001.tif]

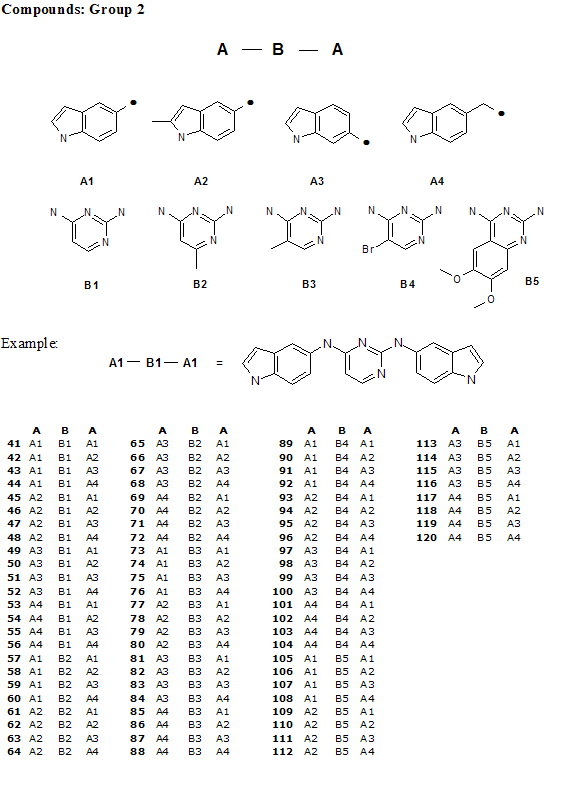

Supplement: Figure S2 — Chemical structures of group 2 potential Rac-GTPase-inhibiting compound formulas theoretically considered for in vitro testing. For Materials and Methods see text S1. (TIF) [file pone.0074924.s002.tif]

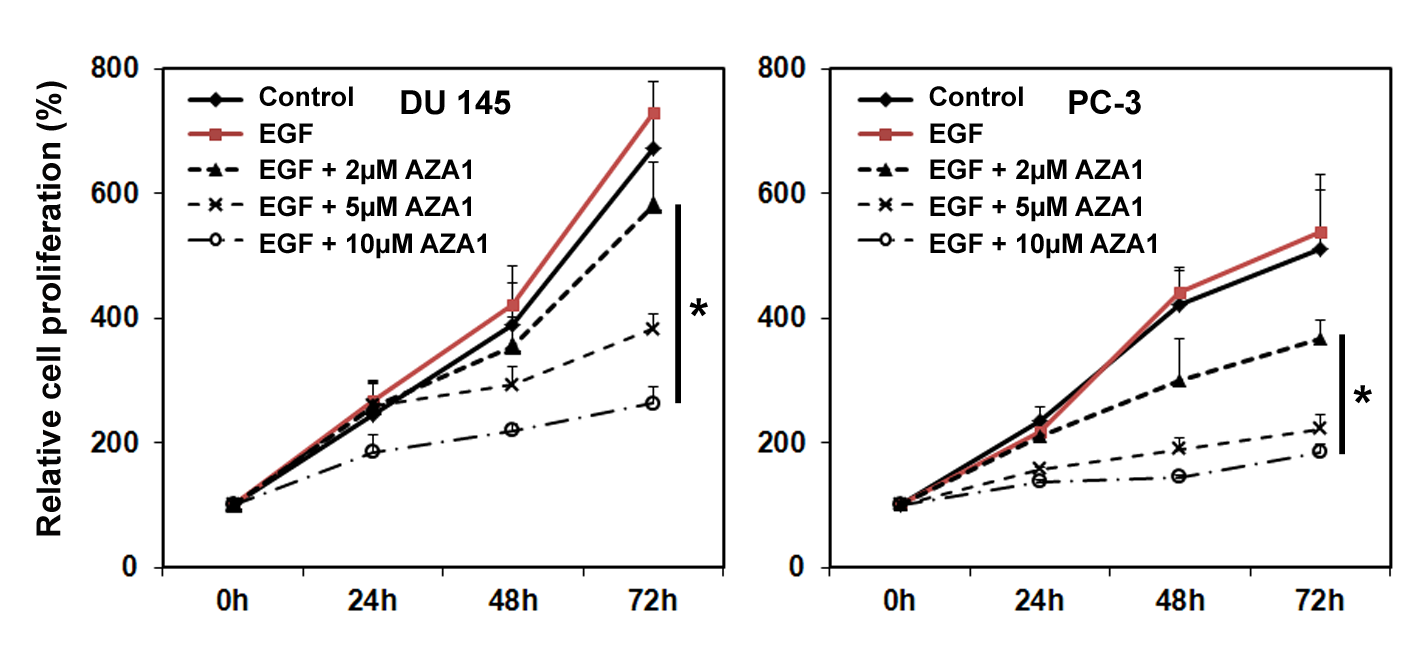

Supplement: Figure S3 — Rac1 and Cdc42 inhibition by AZA1 reduces the proliferation of DU 145 and PC-3 prostate cancer cells. Relative density of cancer cells up to 72 h following treatment with 2, 5, and 10 µM compound AZA1 in EGF-stimulated cancer cells was measured using the WST-1 cell proliferation assay. AZA1 suppresses DU 145 and PC-3 prostate cancer cell proliferation in EGF-stimulated cancer cells in a dose-dependent manner. Means of three independent experiments are shown. *, significantly different from untreated control and EGF-stimulated cells (p<0.05). (TIF) [file pone.0074924.s003.tif]

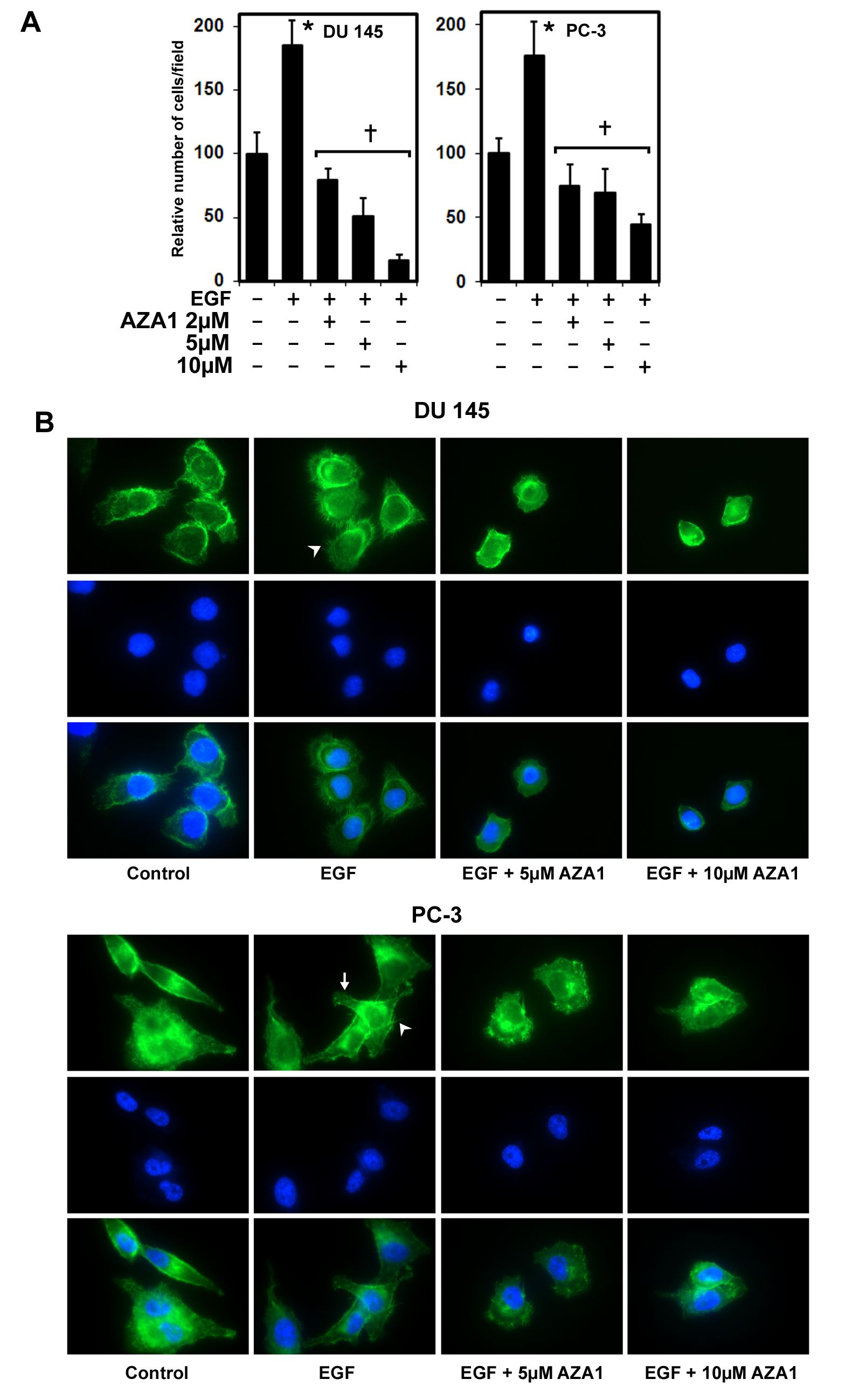

Supplement: Figure S4 — Rac1 and Cdc42 blockade reduces prostate cancer cell migration and affects cytoskeletal dynamics in DU 145 and PC-3 prostate cancer cells. A, Rac1 and Cdc42 blockade reduces prostate cancer cell migration. DU 145 and PC-3 prostate cancer cells were stimulated with 50 ng/ml EGF and treated with 2, 5 and 10 µM AZA1 for 24 h and migrated cancer cells quantified subsequently in vitro. Data were collected from five individual consecutive fields of view (40x) from three replicate Boyden chambers. *, significantly different from control; +, significantly different from control and EGF-stimulated cells. B, Effects of AZA1 treatment on lamellipodia and filopodia formation. DU 145 (upper three panels) and PC-3 (lower three panels) prostate cancer cells were plated on cell culture chambers, stimulated with 50 ng/ml EGF and incubated with 5 and 10 µm AZA1 for 24 h. Paraformaldehyde fixed cells were stained with Atto-488 phalloidin (F-actin, green) and nuclei were counterstained with DAPI (blue). Lowest panel: merge panel. Arrow head indicates filopodia, arrow indicates lamellipodia. AZA1 leads to changes in cellular morphology and suppresses filopodia (DU 145 and PC-3) and lamellipodia (PC-3) formation (magnification, x1000). (TIF) [file pone.0074924.s004.tif]

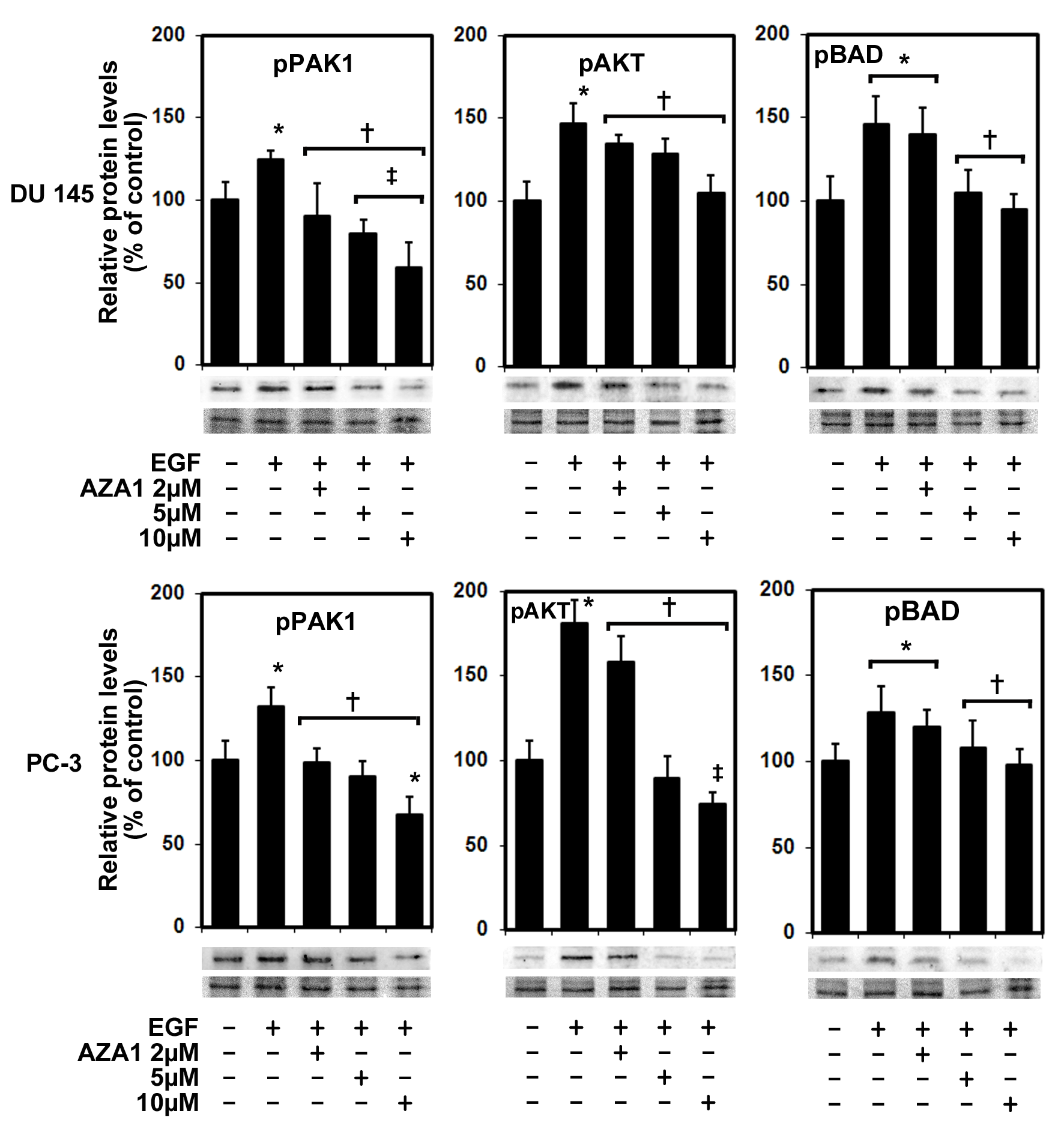

Supplement: Figure S5 — Analysis of PAK-, AKT- and BAD-phosphorylation in EGF-stimulated DU 145 (upper panel) and PC-3 (lower panel) prostate cancer cells following AZA1 treatment. Representative Western blot images and quantification of immunoblots stained with phospho-PAK1/2 (pPAK), phospho-AKT (pAKT) and phospho-BAD (pBAD) antibodies before and after treatment with 2, 5 and 10 µM AZA1 for 24 hours. Rac1/Cdc42 blockade reduces phosphorylation of PAK1, AKT and BAD in prostate cancer cells compared to controls (means of 3 independent experiments). *, significantly different from unstimulated and untreated control; +, significantly different from EGF-stimulated control; ‡, significantly different from unstimulated, untreated control and EGF-stimulated control. (TIF) [file pone.0074924.s005.tif]
