# Supplementary material for: A Rac1/Cdc42 GTPase-Specific Small Molecule Inhibitor Suppresses Growth of Primary Human Prostate Cancer Xenografts and Prolongs Survival in Mice
Source: PLoS One. 2013 Sep 11;8(9):e74924. doi: 10.1371/journal.pone.0074924 (PMC3770583; doi:10.1371/journal.pone.0074924)
Supplement: Table S1 — 21 synthesized compounds tested in vitro for solubility, GTPase activation and effects on cell proliferation. Compound AZA1 was selected for further experiments in vivo. Solubility: Soluble in 30% dimethyl sulfoxide (DMSO) at 1 mM compound concentration: (+), soluble; (−), insoluble or poorly soluble. Toxicity (WST-1 cell proliferation assay): (+), substance tolerable; (−), substance discarded due to toxicity at < 25 µM (concentrations tested: 1−100 µM). Rac-Inhibition: (+), (++) relative inhibition of Rac activity; (−) negligible inhibition of Rac activity. For Materials and Methods see text S1. (DOCX) [file pone.0074924.s006.docx]

| **Compound** | Toxicity | **Rac inhibition** | | **Solubility in 30% DMSO** | Selected compound for *in vivo* experiments |
| --- | --- | --- | --- | --- | --- |
| **Group 1** |  | **4 h** | **24 h** |  |  |
| A1-B1-C1 | **+** | **-** | **-** | **-** |  |
| A2-B1-C1 | **+** | **-** | **-** | **+** |  |
| A3-B1-C1 | **+** | **-** | **+** | **-** |  |
| A4-B1-C1 | **+** | **-** | **-** | **+** |  |
| A2-B2-C1 | **+** | **-** | **+** | **+** |  |
| A2-B3-C1 | **+** | **+** | **+** | **+** |  |
| A1-B4-C1 | **-** | **+** | **+** | **+** |  |
| A2-B4-C1 | **-** | **-** | **+** | **+** |  |
| A1-B5-C1 | **-** | **-** | **-** | **-** |  |
| A2-B1-C2 | **+** | **-** | **-** | **+** |  |
| A1-B2-C2 | **+** | **-** | **-** | **+** |  |
| A2-B2-C2 | **+** | **-** | **-** | **+** |  |
| A3-B3-C2 | **+** | **-** | **-** | **-** |  |
| A4-B5-C2 | **-** | **-** | **-** | **+** |  |
| **Group 2** |  |  |  |  |  |
| A1-B1-A1 | **-** | **+** | **+** | **+** |  |
| A1-B1-A4 | **+** | **-** | **-** | **-** |  |
| A2-B1-A2 (AZA1) | **+** | **++** | **++** | **+** | **+** |
| A4-B1-A3 | **-** | + | **+** | **+** |  |
| A4-B1-A4 | **-** | **-** | **+** | **+** |  |
| A4-B2-A4 | **-** | **-** | **-** | **+** |  |
| A4-B5-A4 | **-** | **-** | **-** | **+** |  |
