# Supplementary material for: A Rac1/Cdc42 GTPase-Specific Small Molecule Inhibitor Suppresses Growth of Primary Human Prostate Cancer Xenografts and Prolongs Survival in Mice
Source: PLoS One. 2013 Sep 11;8(9):e74924. doi: 10.1371/journal.pone.0074924 (PMC3770583; doi:10.1371/journal.pone.0074924)
Supplement: Text S1 — Supplemental Materials and Methods (DOCX) [file pone.0074924.s007.docx]

**Supplemental Text S1**

**Chemical structures of compound candidates.** Chemical structures of diverse potential Rac-GTPase-inhibiting compound formulas which were theoretically considered for testing *in vitro* are shown in Supplemental Figures S1 and S2.

**Synthesized compounds.** We generated 21 chemically diverse potential Rac-inhibiting compound formulas from 120 compound structures (Supplemental Figures S1 and S2), which were then synthesized by SPECS (Delft, Netherlands). Subsequently, all synthesized compounds were tested *in vitro* for solubility, activation and effects on proliferation (WST-1).

**Rac1 activation assays.** Prostate cancer cells were seeded in 6-well plates and starved for 24 h. Cells were incubated with 10 and 20 µM compound for 4 and 24 hours and then stimulated with 50 ng/ml epidermal growth factor (EGF; R&D Systems, Minneapolis, MN) for 90 sec and Rac1 activity was then measured with G-LISA (colorimetric format, Cytoskeleton, Denver, CO) according to the manufacturer’s protocol.

***In vitro* compound selection.** Based on compound solubility, effects on proliferation and inhibition of Rac activation, compound AZA1 was selected for further analysis *in vitro* and *in vivo* (Supplemental Table S1).
